# Oral ingestion of Shiikuwasha extract suppresses diabetes progression in *db/db* mice by preserving β-cell mass

**DOI:** 10.3389/fnut.2023.1336133

**Published:** 2024-01-05

**Authors:** Megumi Kaji, Yukiko K. Kaneko, Stella Amarachi Ihim, Ran Kanoh, Moe Yamamoto, Momoka Yamaguchi, Tomohisa Ishikawa

**Affiliations:** Department of Pharmacology, School of Pharmaceutical Sciences, University of Shizuoka, Shizuoka, Japan

**Keywords:** Shiikuwasha extract, nobiletin, type 2 diabetes, *db/db* mice, pancreatic β-cells, insulin

## Abstract

**Introduction:**

Nobiletin is a polymethoxyflavonoid abundant in citrus peels and has been reported to have various bioactive effects. We have previously reported that nobiletin inhibits endoplasmic reticulum stress-induced apoptosis in the pancreatic β-cell line INS-1 and that continuous subcutaneous administration of nobiletin suppresses the progression of diabetes by protecting β-cells in type 2 diabetic *db/db* mice. In the present study, we investigated effects of oral ingestion of Shiikuwasha extract rich in nobiletin on the pathogenesis of type 2 diabetes in *db/db* mice.

**Materials and methods:**

A Shiikuwasha extract was dissolved in MediDrop sucralose. Twenty-four mice were equally divided in three groups and fed with vehicle or low or high dose of Shiikuwasha extract for 4 weeks. Blood glucose levels, pancreatic β-cell mass, serum insulin levels, pancreatic insulin content, and other biomarkers were measured and compared between the groups.

**Results:**

The group that freely ingested the Shiikuwasha extract containing higher concentration of nobiletin (Shiikuwasha H) showed lower blood glucose levels. At the end of the experiment, the Shiikuwasha H group exhibited improved glucose tolerance, lower serum glycoalbumin levels, and an increase in β-cell area per pancreas compared with the control group. Body weight, food intake, and serum biomarkers related to liver function and lipid metabolism of the Shiikuwasha H group were not different from those of the control group, although water intake of the former was significantly decreased than that of the latter.

**Conclusion:**

Our results suggest that the oral ingestion of Shiikuwasha extract preserves pancreatic β-cell mass in diabetic mice, which might be attributed to ameliorating the progression of diabetes.

## 1 Introduction

Diabetes is a chronic disease characterized by insulin resistance and impaired insulin secretion. Particularly in type 2 diabetes, oxidative and endoplasmic reticulum (ER) stress in pancreatic β-cells caused by glucotoxicity, lipotoxicity, and glucolipotoxicity lead to dysfunction of insulin secretion from pancreatic β-cells. ER stress induces pancreatic β-cell apoptosis, decreasing the number of cells and insulin secretion, which further progress type 2 diabetes (1). Thus, restoring the β-cell mass may prevent the progression of type 2 diabetes.

Nobiletin is a citrus flavonoid that is abundant in the peel of citrus fruits, such as Shiikuwasha (*Citrus depressa* Hayata) and Ponkan (*Citrus reticulata*) (2), and has been reported to have anti-inflammatory (3, 4), anti-tumor (5, 6), hepatitis suppressing (7), anti-obesity (8, 9), and anti-insulin resistance effects (10). We previously demonstrated that nobiletin facilitates glucose-stimulated insulin secretion and inhibits ER stress-induced apoptosis in INS-1D cells, a rat pancreatic β-cell line (11).

*Db/db* mice are a mouse model of type 2 diabetes with obesity known to develop a decrease in β-cell number as diabetes progresses. Hence, *db/db* mice are employed as a suitable model for evaluating the effects on the decrease in β-cell mass in type 2 diabetes. Recently, we showed that the subcutaneous implantation of nobiletin-filled osmotic pumps in *db/db* mice reduces the progression of diabetes and preserves β-cell mass (12). Other groups have also reported that feeding diabetic model mice citrus extract containing nobiletin effectively reduces the onset of diabetes by reducing obesity and insulin resistance (10, 13). In the present study, we aimed to investigate whether oral ingestion of nobiletin-rich Shiikuwasha extracts, which are widely and commercially available, ameliorates the pathogenesis of diabetes through their effects on pancreatic β-cells.

## 2 Materials and methods

### 2.1 Animals

Type 2 diabetes model mice *db/db* (5-weeks-old, 20–30 g, males) were purchased from SLC (Hamamatsu, Japan). The animals were housed under a 12-h light–dark cycle with food *ad libitum*. All experiments using laboratory animals were performed according to the protocols approved by the Institutional Animal Care and Use Committee of the University of Shizuoka (approved number: #166214 and #196383) and the Guidelines for Animal Experiments established by the Japanese Pharmacological Society.

### 2.2 Experimental design and feeding of Shiikuwasha extract

**Figure 1:**
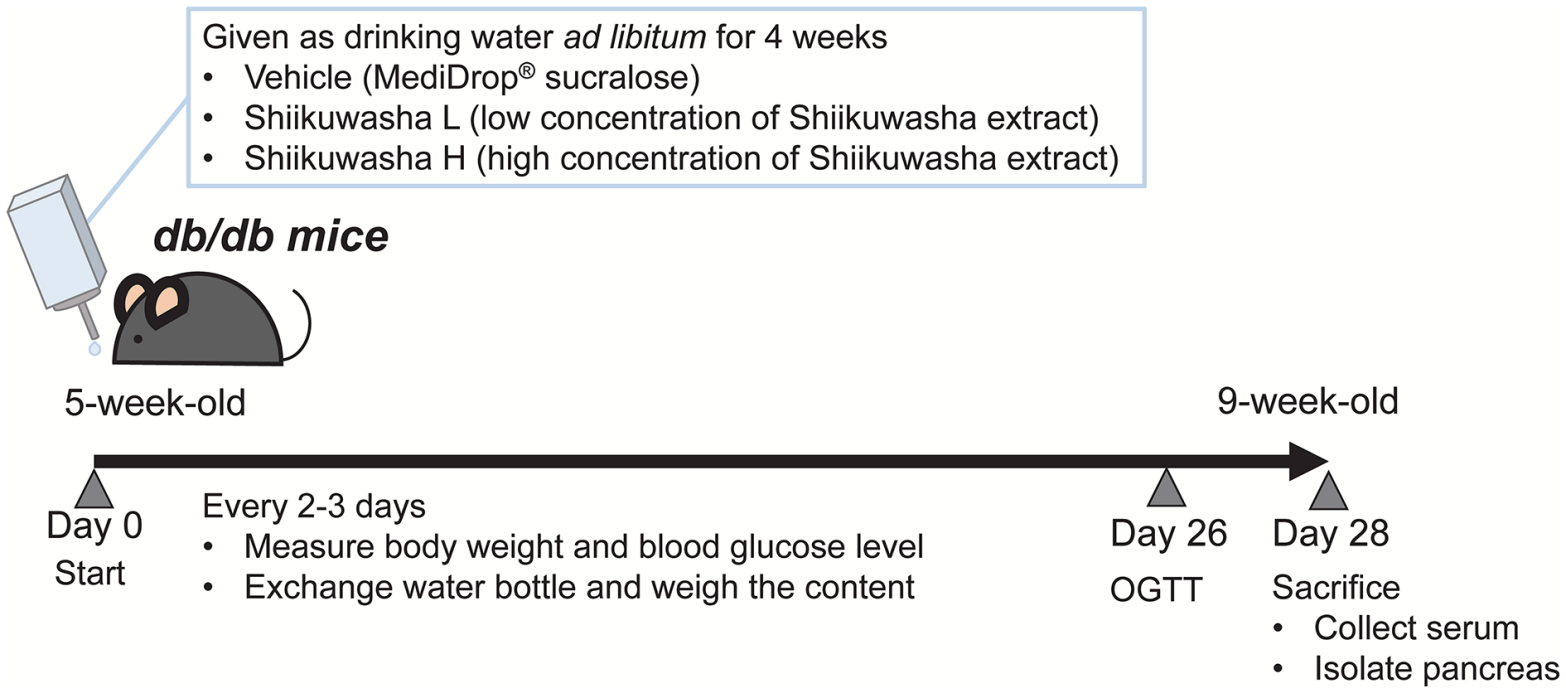
Flowchart of the experiment design.

**Figure 2:**
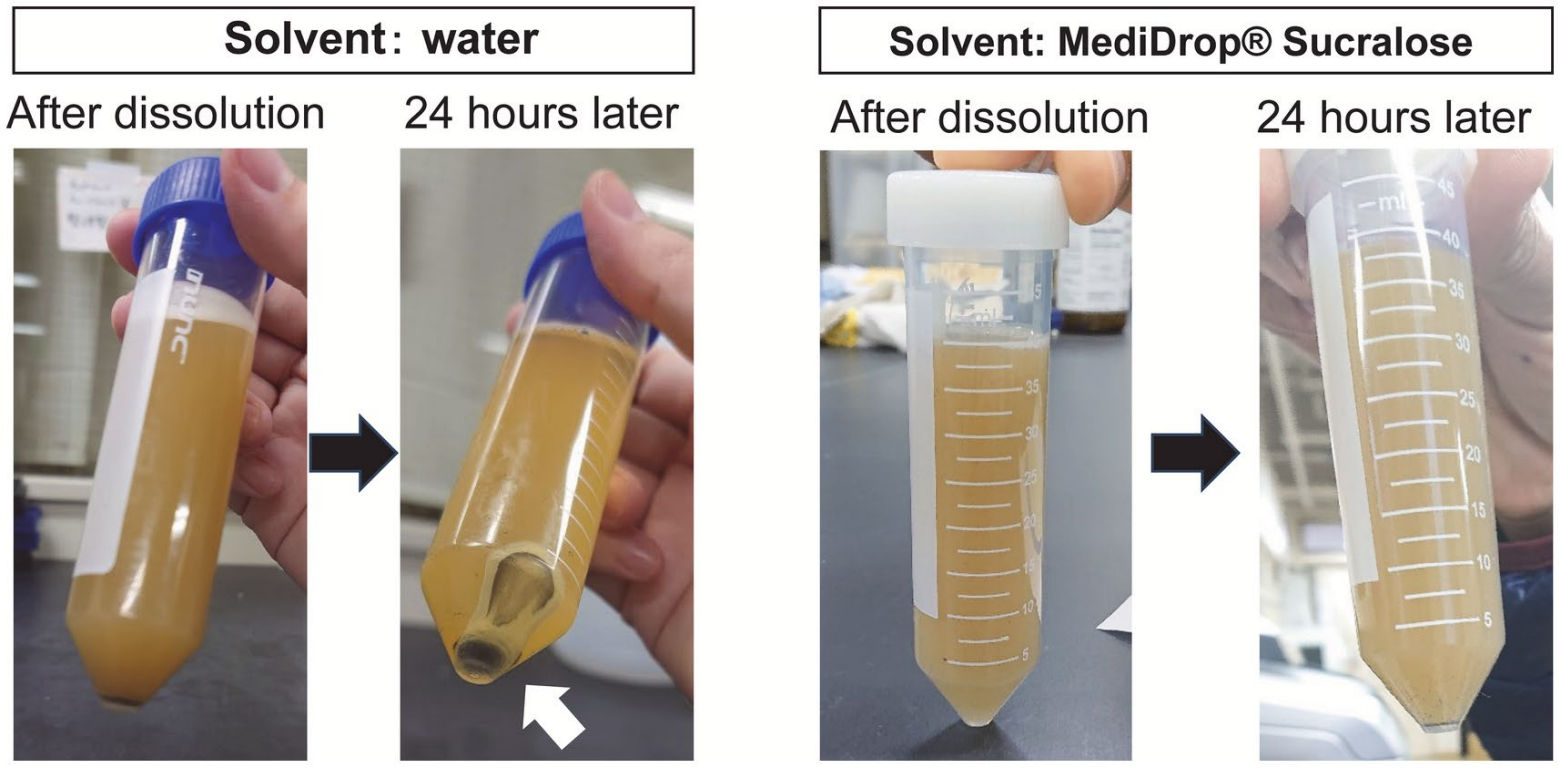
Comparison of the solvents used to dissolve the Shiikuwasha extract. Water and MediDrop® sucralose were used to dissolve the extract. Upon dissolution in aqueous media, precipitates appeared within 24 h, as indicated by the arrows, whereas no precipitates were observed with the use of MediDrop® sucralose.

The flowchart of experimental design is shown in Figure 1. A powdered Shiikuwasha extract (*Citrus depressa* Hayata) rich in nobiletin and tangeretin (7.7 and 3.4%, respectively, as determined by high performance liquid chromatography, Biletin, Arkray, Japan) was used in the experiments. Because the Shiikuwasha extract was insoluble in water and had an intense bitter taste, mice were unable to ingest enough of the Shiikuwasha extract suspended in water. In addition, it was imperative that the extract exhibited sustained and stable resolution without precipitating events over an extended period. Therefore, to enhance the Shiikuwasha extract ingestion, we employed MediDrop® sucralose (ClearH_2_O, Westbrook, ME, United States) for the experiments to reduce the bitter taste and completely suspend the Shiikuwasha extract (Figure 2). Referring previous reports showing that the oral administration of 20–50 mg/kg nobiletin increased the blood levels of the flavonoid to sub micromolar concentrations (14, 15), low (0.40 ± 0.010 mg/mL) and high (5.01 ± 0.185 mg/mL) concentrations of the Shiikuwasha extract dissolved in the solvent were prepared considering body weight of each mouse. Mice were freely ingested the solvent without or with low or high concentrations of the Shiikuwasha extract. The solvent intake was determined by measuring changes in the weight of water bottle every 2 or 3 days. The doses of the Shiikuwasha extract calculated from the solvent intake were 11.0 ± 0.200 and 69.5 ± 1.54 mg/kg/day in the low dose-ingested (Shiikuwasha L) and high dose-ingested groups (Shiikuwasha H), respectively.

Mice were weighed and blood glucose levels were measured 2 days before the start of the experiment. Twenty-four mice were equally and randomly divided into three groups: control, Shiikuwasha L, and Shiikuwasha H groups. Body weight and water intake were measured once a week, and food intake was measured on day 9. Mice were fasted for 1 h prior to the measurements. Blood glucose levels were measured using an ACCU-CHECK ST METER (Roche Diagnostics, Basel, Switzerland) or STAT STRIP XPRESS 900 (Siemens, Germany). An oral glucose tolerance test (OGTT) was performed 3 weeks after the start of the experiment. At the end of the 4-week treatment period, blood samples were collected from the inferior vena cava and the pancreas and white adipose tissue around the testis were isolated under isoflurane anesthesia. Serum was used for the measurement of biomarkers and the pancreas was used for the measurement of pancreatic insulin content and β-cell mass.

### 2.3 Oral glucose tolerance test

On day 26, mice were moved to metabolic cages and fasted for 12 h prior to OGTT. Blood was collected from the tail vein and blood glucose levels were measured using a glucose meter at 0, 15, 30, 60, 90, and 120 min after oral glucose administration (2 g/kg).

### 2.4 Measurement of pancreatic insulin content, serum insulin, and other biomarkers

The isolated pancreas was minced in 2 mL of acidic ethanol solution (2% HCl in 75% EtOH) and homogenized using an ULTRA-TURRAX disperser (IKA, Germany) under ice-cold conditions. The total volume of samples was then made up to 5 mL and left overnight at 4°C. Samples were vortexed, centrifuged (4°C, 15,000 *g*, 10 min), and the supernatant was collected as the samples for pancreatic insulin content measurement. Blood collected from the inferior vena cava was left at room temperature for 30 min followed by overnight incubation at 4°C. Blood samples were then centrifuged (4°C, 1,000 *g*, 30 min), and the supernatants were collected as serum samples. The insulin concentration in the samples was quantified using a mouse insulin ELISA Kit (Morinaga Institute of Biological Sciences, Inc., Yokohama, Japan). Other biomarkers were measured by Oriental Yeast Co. LTD (Tokyo, Japan).

### 2.5 Measurement of β-cell mass

The isolated pancreases were fixed overnight at 4°C in 4% paraformaldehyde/phosphate-buffered saline (PBS). They were then incubated in 10% sucrose/PBS, followed by 15% sucrose/PBS for 4 h at 4°C. The tissues were further dehydrated by placing in 20% sucrose/PBS overnight at 4°C. The pancreas was embedded in Tissue-Tek™ O.C.T. compound (Sakura Finetek, Nagano, Japan) and cryosectioned into 5-μm slices. Thin sections were blocked with 3% bovine serum albumin/PBS for 15 min and incubated with a primary antibody (guinea pig anti-insulin antibody, diluted 1:500; Fitzgerald Industries International, MA, United States) overnight at 4°C. After washing, a secondary antibody (goat anti-guinea pig antibody Alexa Fluor 555, diluted 1:200; Invitrogen, United States) was added and incubated for 1 h at room temperature. The sections were mounted with Fluoro-KEEPER Antifade Reagent, Non-Hardening Type with DAPI (Nacalai Tesque, Kyoto, Japan), and observed and analyzed using a fluorescence microscope BZ-X700 (KEYENCE, Osaka, Japan). Wide visual images of the pancreas were obtained using the automatic stitching function.

### 2.6 Statistical analysis

Results in the text and figures are presented as means ± standard deviation (SD) or means ± standard error of the mean (SEM). Statistical analysis was performed by the Dunnett’s test using software *R*, and a *p* value <0.05 was considered as statistically significant.

## 3 Results

### 3.1 Effects of the Shiikuwasha extract on body weight, food and water intake, and casual blood glucose

**Figure 3:**
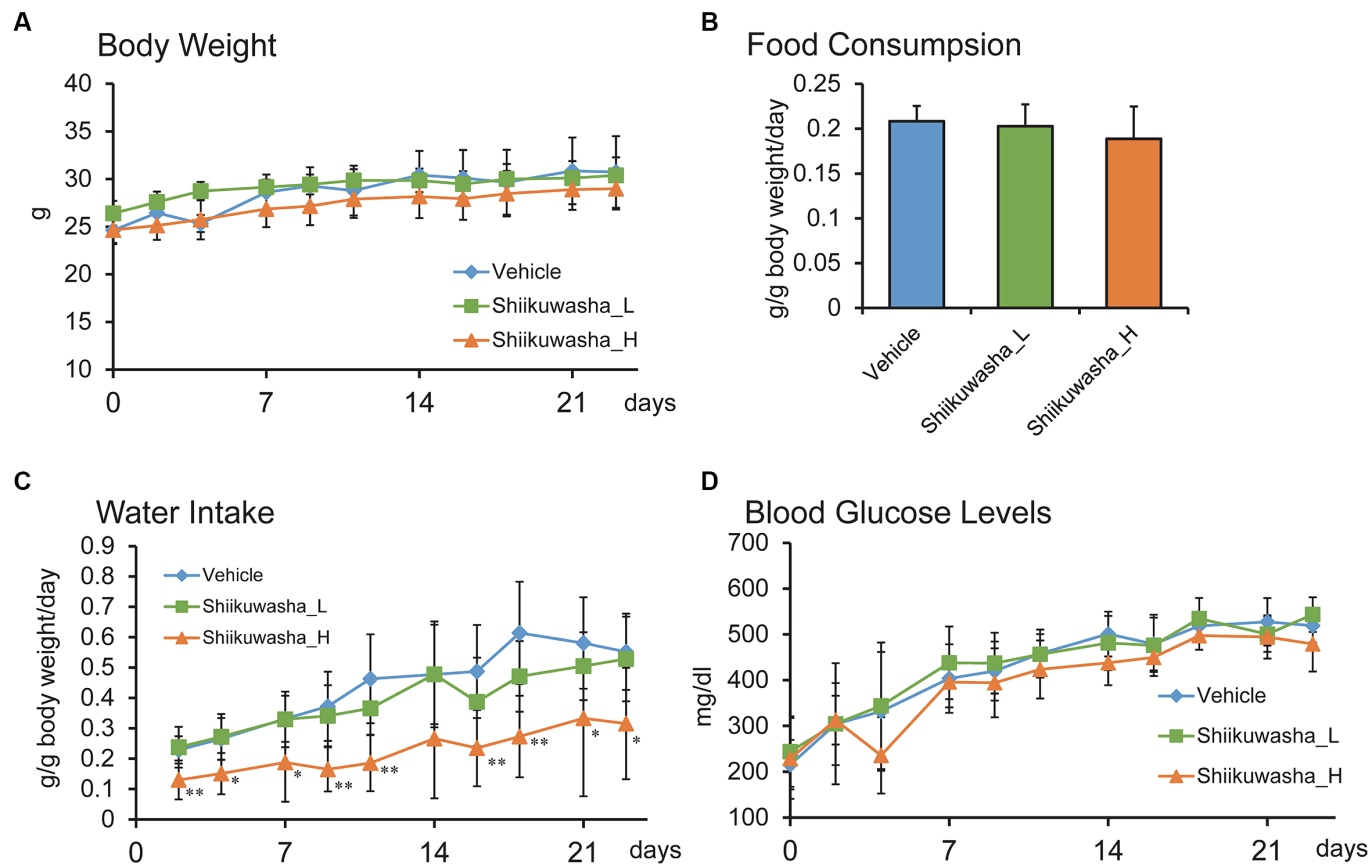
Effects of the Shiikuwasha extract on body weight, water and food intake, and casual blood glucose levels in *db/db* mice. The mice were divided into three groups, i.e., vehicle-fed group (Vehicle), low-dose Shiikuwasha extract-fed group (Shiikuwasha_L), and high-dose Shiikwasha extract-fed group (Shiikuwasha_H). Changes in body weight during the treatment period **(A)**, water intake **(C)**, and casual blood glucose levels **(D)** are shown (*n* = 8). Food consumption on day 9 is shown in panel B (*n* = 4–6). Data are presented as means ± SD **(A,C,D)** or means ± SEM **(B)**. ^*^p < 0.05.

The effects of the 4-week oral ingestion of the Shiikuwasha extracts on body weight, water intake, and casual blood glucose levels were examined. During the experimental period, no difference in body weight was identified between the control vehicle group and the Shiikuwasha L and H groups (Figure 3A). Although no significant difference was found in food consumption between three groups (Figure 3B), the mean amount of water intake was lower in the Shiikuwasha H group (Figure 3C) compared with in the control group. We also examined changes in casual blood glucose levels in *db/db* mice after 3-week treatment with the Shiikuwasha extracts. Blood glucose levels of the Shiikuwasha H group tended to be lower than those of the control and Shiikuwasha L groups (Figure 3D).

### 3.2 Effects of the Shiikuwasha extract on glucose tolerance

**Figure 4:**
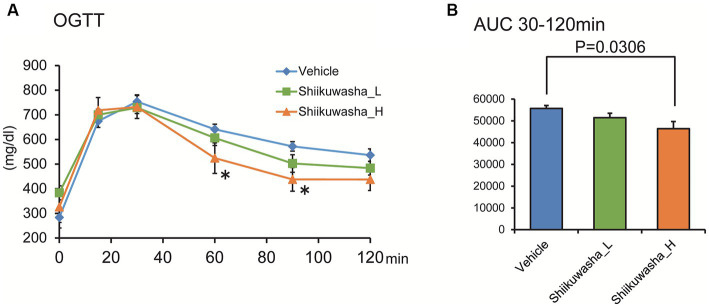
Effect of the Shiikuwasha extract on the oral glucose tolerance test in *db/db* mice. **(A)** Three weeks after the start of treatment, an oral glucose tolerance test (OGTT, glucose 2 g/kg, p.o.) was performed after 12-h fasting. **(B)** The area under the curve was calculated from the blood glucose levels between 30 and 120 min after glucose administration. Data are presented as means ± SEM (*n* = 8). ^*^p < 0.05.

After a 3-week administration of the Shiikuwasha extract to *db/db* mice, an OGTT was performed to determine its effects on glucose tolerance. Fasting blood glucose levels and acute blood glucose elevation after glucose administration did not differ among three groups. However, blood glucose levels of the Shiikuwasha H group were significantly lower 60 and 90 min after glucose administration compared with those of the vehicle control group (Figure 4A). Although the total area under the curve (AUC) between 0 and 30 min did not differ between three groups, the AUC between 30 and 120 min was significantly lower in the Shiikuwasha H group than in the control group (Figure 4B). These results suggested that the Shiikuwasha extract improved glucose tolerance in *db/db* mice.

### 3.3 Effects of the Shiikuwasha extract on the serum insulin level, pancreatic insulin content, and other biomarkers

**Figure 5:**
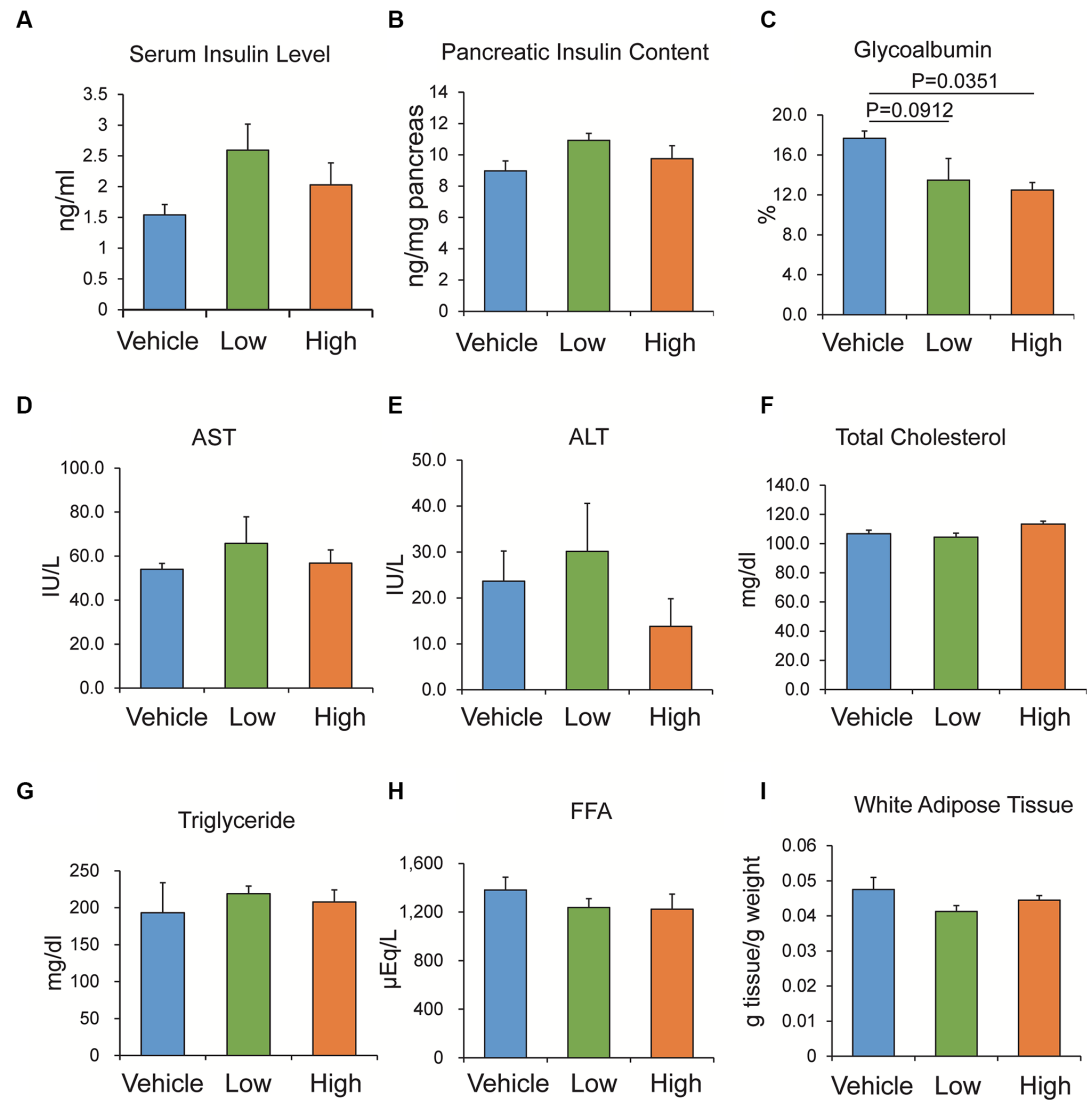
Effects of the Shiikuwasha extract on serum insulin levels, pancreatic insulin content, and other biomarkers in *db/db* mice. After 4-week administration of vehicle or low- or high-concentration of Shiikuwasha extract to *db/db* mice, serum was collected to measure serum insulin (**A**, *p* = 0.234 and *p* = 0.435 vs. control in Shiikuwasha_L and Shiikuwasha_H groups, respectively), glycoalbumin **(C)**, aspartate aminotransferase (AST, **D**), alanine aminotransferase (ALT, **E**), total cholesterol **(F)**, triglyceride **(G)**, and free fatty acid (FFA, **H**). **(B)** The pancreatic insulin content was determined by extracting insulin from the pancreas using an acid-ethanol extraction method. *p* = 0.176 and *p* = 0.697 vs. control in Shiikuwasha_L and Shiikuwasha_H groups, respectively. **(I)** White adipose tissue around the testis was isolated from *db/db* mice and weighed to calculate adipose tissue weight per body weight. **(I)** Data are presented as means ± SEM (*n* = 4–8).

Non-fasting serum insulin levels and pancreatic insulin content were measured after the 4-week administration of the Shiikuwasha extract to *db/db* mice. Although no significant effect was identified on the serum insulin level and pancreatic insulin content in the Shiikuwasha extract-fed and vehicle-fed control mice, the administration of the Shiikuwasha extract tended to increase the serum insulin level and pancreatic insulin content (Figures 5A,B). Furthermore, the plasma glycoalbumin level was significantly lower in the Shiikuwasha H group and tended to be lower in the Shiikuwasha L group, compared to those in the vehicle control group (Figure 5C). However, no differences were observed in various serum values related to liver function and lipid metabolism after the 4-week administration of the Shiikuwasha extract to *db/db* mice (Figures 5D–H). Additionally, no significant differences were found in adipose tissue weight around testis between the Shiikuwasha extract-fed and the vehicle control mice (Figure 5I). Other biomarkers, except glycoalbumin, were not affected by the administration of the Shiikuwasha extract.

### 3.4 Effects of the Shiikuwasha extract on islet morphology

**Figure 6:**
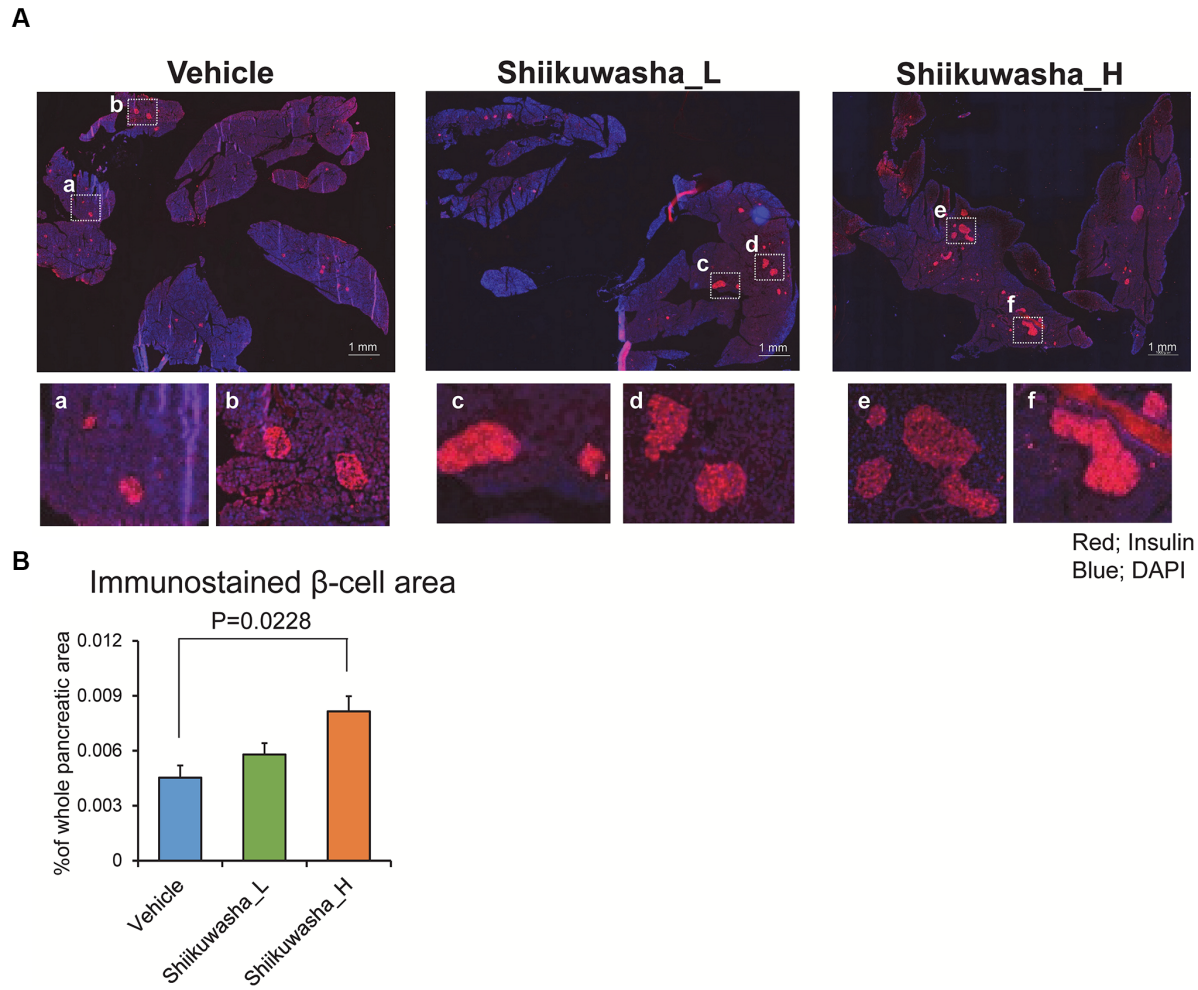
Effects of the Shiikuwasha extract on insulin-positive area in the pancreas of *db/db* mice. **(A)** After the 4-week treatment, pancreatic tissue was isolated from *db/db* mice, fixed in 4% paraformaldehyde, embedded in Tissue Tek™ O.C.T. compound, and cryosectioned into 5-μm sections. The sections were stained with an anti-insulin antibody (red) and 4′,6-diamidino-2-phenylindole (DAPI, blue). Scale bar = 1 mm. **(B)** The insulin-positive area per pancreas was quantified by immunostaining with an anti-insulin antibody (red) and DAPI (blue). Data are presented as means ± SEM (*n* = 4). ^*^p < 0.05.

To investigate the effect of the Shiikuwasha extract on β-cell mass, the pancreatic β-cell area was measured by immunostaining of cryosections of pancreases. Numerous large islets were observed in the Shiikuwasha H group. Analysis of the insulin-positive area per unit of pancreatic area showed that the insulin-positive pancreatic β-cell area was significantly increased in the Shiikuwasha H group compared to the vehicle control group (Figures 6A,B). These results suggested that the Shiikuwasha extract had an ameliorating effect on diabetes in *db/db* mice, due to the preservation of pancreatic β-cells.

## 4 Discussion

Nobiletin, a natural polymethoxyflavonoid and component of citrus extract, reportedly possesses anti-tumor (5, 6) and anti-inflammatory (3, 4) properties, as well as anti-obesity and insulin resistance-ameliorating effects in type 2 diabetes (9, 10). We have previously reported that nobiletin and nobiletin-containing citrus extract have potent inhibitory effects on glucose-induced insulin secretion and on ER stress-induced apoptosis in INS-1D cells, a rat-derived pancreatic β-cell line (11). Recently, we additionally found that the continuous administration of nobiletin using a subcutaneously implanted osmotic pump prevented β-cell loss in *db/db* diabetic mouse models (12). In the present study, we analyzed the effects of oral administration of the nobiletin-containing Shiikuwasha extract on the progression of diabetes and β-cell mass in *db/db* mice to determine whether such administration maintained β-cell mass.

The intake of the Shiikuwasha extract may vary when fed to mice because of its bitter taste (16) and precipitation due to water insolubility when mixed with drinking water. To overcome this issue, we first tried to use a commercially available bitter-masking powder mixture (Benecoat BMI40; Kao Corp., Japan) to mask the bitterness and prevent the precipitation (data not shown). Although Benecoat allowed the Shiikuwasha extracts to mix evenly with water, the bitterness might not have been fully masked; the intake of water in the extract-treated group dropped to approximately 23% of that in the vehicle control group. In contrast, Medidrop was a more appropriate solvent, as it allowed uniform mixing of the Shiikuwasha extract and maintained water consumption in the extract-fed groups approximately 50% of that in the vehicle control group (Figure 3C). Although the amount of water consumption was lower in the Shiikuwasha H group than in the control group, the amount of food intake remained unchanged (Figure 3B), suggesting that Medidrop enabled mice to receive nobiletin continuously and that water and food intake themselves have little effect on the progression of diabetes. The intake of water has been shown to be correlated with reduced risk of developing diabetes (17). It is therefore possible that the decreased water consumption in the Shiikuwasha H group might have weakened the amelioration of diabetes by the Shiikuwasha extract. On the other hand, since increased thirst is one of the main symptoms of diabetes, the reduced water consumption in the Shiikuwasha H group might have been due to improvement in diabetes.

The actual amount of nobiletin ingested by the mice and the blood nobiletin concentration were not measured in the present study. A previous study has reported that upon oral administration of 20 mg/kg nobiletin to rat, the plasma nobiletin concentration was elevated to 1 μM (15). Based on this report, we estimated that the plasma nobiletin concentration in the present study would be elevated to approximately 0.5 or 5 μM in the Shiikuwasha L and Shiikuwasha H groups, respectively.

The Shiikuwasha extract used in the present study contained 3.4% tangeretin and 7.7% nobiletin. Tangeretin, similar to nobiletin, has been reported to have antidiabetic effects (18). Therefore, other polymethoxyflavonoids such as tangeretin may also be involved in the antidiabetic effects observed in the present study. However, our findings suggest that nobiletin is involved in the antidiabetic effect, since these effects, such as the β-cell preservation observed in the Shiikuwasha H group, are similar to our previous results using nobiletin alone (12).

In the OGTT conducted 3 weeks after administration of the Shiikuwasha extract, the glucose tolerance of the Shiikuwasha H group was improved compared to that of the vehicle group. Serum insulin levels and pancreatic insulin content, measured using serum and pancreatic samples collected after 4-week treatment, tended to be elevated in the Shiikuwasha extract-treated group. Moreover, serum glycoalbumin levels, which reflect blood glucose levels for approximately 2 weeks, were significantly lower in the Shiikuwasha H group, suggesting that Shiikuwasha extract supplementation improves glucose tolerance, leading to long-term improvement in blood glucose levels.

The pancreatic β-cell area measured with immunostaining was significantly larger in the Shiikuwasha H group than in the vehicle group. Furthermore, although no difference was observed in blood glucose levels after a 12-h fasting, the time required to recover basal levels from the transient increase in blood glucose levels caused by glucose loading was shortened in the group treated with the Shiikuwasha extract. In contrast, the weight of white adipose tissue around testis per body weight did not change after the administration of the Shiikuwasha extract. These results suggest that pancreatic β-cells in the Shiikuwasha extract-fed group secretes a sufficient amount of insulin to lower the blood glucose levels, possibly due to the inhibition of β-cell loss by the Shiikuwasha extract. Since nobiletin has been shown to improve insulin resistance (10), the Shiikuwasha extract may also ameliorate insulin resistance. There are also several reports showing that oral ingestion of other food ingredients improves the pathophysiology of type 2 diabetes by improving insulin resistance (19, 20).

However, in the present study, the levels of several serum biomarkers related to lipid metabolism and liver function, as well as adipose tissue weight, were not altered by the ingestion of the Shiikuwasha extract, suggesting little effect on insulin resistance. Lee et al. have reported that 1.5% (w/w), but not 1% (w/w), Shiikuwasha extract in experimental diet for 5 weeks improved liver function and lipogenesis accompanied with decreases in body weight gain, visceral fat pad weight, and plasma triglyceride levels in high fat diet-induced obese mice (21), which is expected to ameliorate insulin resistance. In the present study, however, we observed no significant changes in biomarkers related to lipid metabolism or liver function. This discrepancy might be due to difference in the dose of Shiikuwasha extract. The dose of Shiikuwasha extract used in the study by Lee et al. (21) is calculated to be approximately 1.54 g/kg/day even when fed with 1% (w/w) Shiikuwasha extract, which showed no significant effects. Thus, the dose used in the present study, i.e., 10 or 70 mg/kg/day, might be lower than that used in the study by Lee et al. (21). Alternatively, the discrepancy might be due to difference in the models, i.e., *db/db* mice in the present study and high fat diet-induced obese mice in the study by Lee et al. (21). In *db/db* mice, faster diabetes progression associated with marked β-cell dysfunction is observed, compared with in high fat diet-induced obese mice. Thus, β-cell function might have more significant impact on diabetes pathogenesis in *db/db* mice. This notion may be supported by a previous report with resveratrol, a food-derived ingredient, in which it improved glucose tolerance via protecting β-cells without ameliorating insulin resistance in *db/db* mice (22). It is noted that Shiikuwasha extract could show antidiabetic effects via protecting β-cells without affecting biomarkers of lipid metabolism and liver function.

Recently, a human study has reported that rehabilitation with Shiikuwasha extract supplementation improves muscle composition in the nonparetic thigh in subacute stroke patients (23). These findings are in accordance with a rat study which showed that muscle atrophy induced by dexamethasone in aged rats is improved by dietary supplementation with Shiikuwasha extract (24). Thus, it is indicated that the effects of Shiikuwasha extract observed in rodents would be observed in humans as well. The antidiabetic properties of Shiikuwasha extract may have potential for future use in human clinical and preventive medicine.

In summary, we found that the ingestion of Shiikuwasha extract for 4 weeks in *db/db* mice suppressed β-cell loss and inhibited the progression of diabetes. These effects may be attributed to the protective effect of nobiletin on β-cells, which was demonstrated by our previous reports (11, 12). Nobiletin-rich Shiikuwasha extract is expected to be effective in suppressing the progression of diabetes associated with decreased β-cell mass.

## Data availability statement

The original contributions presented in the study are included in the article/supplementary material, further inquiries can be directed to the corresponding author.

## Ethics statement

The animal study was approved by Institutional Animal Care and Use Committee of the University of Shizuoka (approved number: #166214 and #196383). The study was conducted in accordance with the local legislation and institutional requirements.

## Author contributions

MK: Investigation, Methodology, Writing – review & editing, Formal Analysis, Writing – original draft. YK: Conceptualization, Funding acquisition, Investigation, Methodology, Project administration, Writing – original draft, Writing – review & editing. SI: Investigation, Methodology, Writing – review & editing. RK: Investigation, Methodology, Writing – review & editing. MoeY: Writing – review & editing, Investigation, Methodology. MomY: Data curation, Resources, Writing – review & editing. TI: Funding acquisition, Supervision, Writing – review & editing, Writing – original draft.
